# Molecular Imaging of Coronary Plaque Vulnerability Using 18F-Fluorocholine PET-MRI in Patients with Coronary Artery Disease: Validation with Optical Coherence Tomography

**DOI:** 10.3390/jcm14248708

**Published:** 2025-12-09

**Authors:** Jochem A. J. van der Pol, Braim Rahel, Yvonne J. M. van Cauteren, Rik P. M. Moonen, Joan G. Meeder, Suzanne C. Gerretsen, Mueez Aizaz, Claudia Prieto, René M. Botnar, Jan Bucerius, Herman van Langen, Joachim E. Wildberger, Robert J. Holtackers, M. Eline Kooi

**Affiliations:** 1 Department of Radiology & Nuclear Medicine, Maastricht University Medical Center, 6229 HX Maastricht, The Netherlandseline.kooi@mumc.nl (M.E.K.); 2 CARIM Cardiovascular Research Institute Maastricht, Maastricht University, 6229 ER Maastricht, The Netherlands; 3 Department of Cardiology, VieCuri Medical Centre for Northern Limburg, 5912 BL Venlo, The Netherlands; brahel@viecuri.nl (B.R.); hvanlangen@gmail.com (H.v.L.); 4 Department of Cardiology, Maastricht University Medical Center, 6229 HX Maastricht, The Netherlands; 5 Image Guided Therapy, Philips Healthcare, 5648 BC Best, The Netherlands; 6 School of Biomedical Engineering and Imaging Sciences, King’s College London, London WC2R 2LS, UK; 7 Escuela de Ingeniería and Instituto de Ingeniería Biológica y Médica, Pontificia Universidad Catolica de Chile, Santiago 8320000, Chile; 8 Millenium Institute for Intelligent Healthcare Engineering iHEALTH, Santiago 7820436, Chile; 9 Division of Nuclear Medicine, Department of Radiology, Medical University of Graz, 8010 Graz, Austria

**Keywords:** fluorocholine, PET-MRI, optical coherence tomography, plaque imaging, atherosclerosis

## Abstract

**Background/Objectives:** 18F-fluorocholine is a positron emission tomography (PET) tracer earlier found to be a marker of macrophage content in carotid plaques. We aimed to assess the feasibility of 18F-choline PET-MRI to non-invasively localize vulnerable coronary plaques, using optical coherence tomography (OCT) as a reference standard. **Methods:** Patients with recent myocardial infarction who were scheduled for a secondary angiography of a non-culprit vessel underwent 18F-fluorocholine coronary PET-MRI. Subsequently, OCT was performed during the secondary angiography. Maximum target-to-background (TBRmax) values of 18F-fluorocholine uptake were determined in two vessel sections that contained either vulnerable or stable plaques as defined by OCT. The OCT-based definition of a vulnerable plaque was a fibrous cap thickness < 70 µm. To enhance the detectability of coronary plaques using PET, three different motion-correction strategies were used: multigate respiratory gating motion correction (MRG-MOCO), extended MR-based motion correction (eMR-MOCO), and extended MR-based motion correction with ECG gating (eMR-MOCO-ECG). **Results:** Fifteen patients were included in this study. One patient needed to be excluded due to extravasation of the tracer. In another patient, no region with only a stable plaque could be identified. TBRmax values were as follows for three different reconstructions in vulnerable versus stable plaques: MRG-MOCO: mean TBRmax 1.45 vs. 1.35, *p* = 0.52 (*n* = 13); eMR-MOCO: mean TBRmax 1.47 vs. 1.27, *p* = 0.26 (*n* = 11); eMR-MOCO-ECG: mean TBRmax 1.49 vs. 1.26, *p* = 0.21 (*n* = 11). **Conclusions:** 18F-fluorocholine uptake in vulnerable atherosclerotic plaques in coronary arteries was not significantly different from uptake in stable plaques, even though advanced motion-correction methods were applied. That may be caused by multiple factors, such as small coronary plaque size, tracer biology, or remaining cardiac motion.

## 1. Introduction

Atherosclerosis is a progressive, chronic, inflammatory disease with systemic manifestations affecting large- and medium-sized arteries, leading to plaque formation and arterial narrowing. Such plaques can erode or even suddenly rupture, thereby potentially causing a thrombotic occlusion and obstructing the blood flow. The majority of myocardial infarctions (MI) are caused by such thrombotic events [1]. Although vulnerable plaques have certain pathological characteristics, such as positive remodeling, micro-calcification, and a large necrotic core, identification of vulnerable plaques remains challenging [2,3].

Previously, it was shown that implementing cardiac magnetic resonance (CMR) or computed tomography angiography (CTA) in the diagnostic process in NSTEMI patients is a safe gatekeeper for (therapeutic) invasive coronary angiography (ICA) [4]. Regardless, the risk of recurrent MI remains ≈10% within the first year and 5% in each of the subsequent 4 years [5,6]. This is most likely an underestimation, because 17–26% of recurrent plaque ruptures and MI remain clinically silent [7]. The high recurrence rate could be attributable to inaccurate angiographic identification of vulnerable plaques, resulting in inadequate treatment [8,9,10]. The situation is worsened by the fact that vulnerable plaques tend to occur at multiple coronary sites in ≈40% of the patients [10]. Furthermore, it is known that ‘high-risk’ lesions that are anatomically unrelated to the initial event are often responsible for recurrent ischemic events [10,11]. Thus, performing angioplasty of a single lesion may be an insufficient preventive measure.

Non-invasive imaging techniques, such as positron emission tomography (PET), allow studying the natural behavior of plaques longitudinally. Nowadays, hybrid PET-MRI scanners combine the advantages of molecular imaging using PET with the superior soft tissue contrast of magnetic resonance imaging (MRI) [12]. Hybrid PET-MRI also provides a unique opportunity to apply MRI-based non-rigid respiratory motion correction on both MRI and PET images [13].

The radioactive PET tracer 18F-fluorodeoxyglucose (FDG) is mostly used in oncology but has also proven its value in imaging inflammatory changes in the arterial wall. However, FDG remains a more nonspecific inflammation tracer and has several drawbacks in coronary imaging, e.g., physiological uptake in the myocardium. Recent evidence suggests that other, more specific tracers may be used for metabolic imaging of cell activation, especially in macrophages [14]. Earlier studies demonstrated the feasibility of radiolabeled choline for imaging atherosclerosis, showing 18F-fluorocholine PET is able to identify the inflammatory regions in symptomatic carotid artery plaques, with a significant correlation between tracer uptake and macrophage content on histology [15].

Optical coherence tomography (OCT) is an invasive imaging technique that provides detailed images of coronary plaques with a 10–20 micrometers resolution, which is ≈50–100 times higher than what can be achieved using MRI or intravascular ultrasound (IVUS). Due to its superior spatial resolution, OCT can identify a key feature of coronary plaque vulnerability, i.e., the presence of a thin fibrous cap [16]. OCT already proved its usefulness in numerous studies and is therefore considered a reference standard [16,17,18].

In the present feasibility study, we will investigate whether non-invasive 18F-fluorocholine PET-MRI can be used to identify vulnerable atherosclerotic plaques in the coronary arteries. A PET-based imaging marker for plaque vulnerability in the coronary arteries could potentially be used for various purposes, such as to guide clinical decisions or to serve as a marker to investigate the effectiveness of new pharmaceuticals, such as anti-inflammatory medication, on plaque stability. We will test the hypothesis that vulnerable plaques show a locally increased uptake of 18F-fluorocholine on PET compared to stable plaques using invasive OCT as a reference standard.

## 2. Materials and Methods

### 2.1. Study Population

Subjects who presented with non-ST elevation myocardial infarction (NSTEMI) at the VieCuri hospital in Venlo, underwent percutaneous coronary intervention (PCI) of the culprit vessel, and were diagnosed with multivessel coronary artery disease and subsequently scheduled for a second PCI, were recruited for study participation. NSTEMI was defined as ischemic symptoms with elevated cardiac enzymes (Troponin T/I, creatin kinase-MB), however, in the absence of ST-segment elevations in the ECG. Exclusion criteria were conservatively managed patients not scheduled for PCI, ongoing severe ischemia requiring immediate PCI, hemodynamic instability, severe heart failure (Killip Class ≥ III), chest pain highly suggestive of non-cardiac origin, suspicion or evidence of acute aortic dissection, acute pulmonary embolism, acute pericarditis, life-threatening arrhythmias at the cardiac emergency department or before presentation, tachycardia (>100 bpm), angina pectoris secondary to anemia, untreated hyperthyroidism or severe hypertension (>200/110 mmHg), moderate to severe aortic or mitral valve stenosis, pregnancy, and breast feeding and contra-indications for MRI, including metal implants, cardiac implantable devices, claustrophobia, renal failure, and allergy to gadolinium-containing contrast media. This study was approved by the local ethics committee (METC162043/NL58752.068.16) and conducted according to the declarations of Helsinki. All subjects were 18 years or older and provided written informed consent.

### 2.2. PET-MR Imaging

After the first PCI procedure in the acute setting and within 72 h of the scheduled second PCI procedure, hybrid PET-MRI was performed on a fully integrated combined 3 Tesla PET-MRI system (Biograph mMR; Siemens Healthineers, Erlangen, Germany) at the Maastricht University Medical Centre. Subjects were scanned in a headfirst supine position using a 6-channel body matrix and the 12-channel spine radiofrequency coils. First, an MRI-based attenuation map (µ-map) was acquired during an end-expiration breath-hold [18]. Relevant parameters of this Dixon-based µ-map include the following: field-of-view (FOV) = 599 × 271 × 408 mm^3^, acquired resolution = 2.1 × 2.1 × 2.6 mm^3^, flip angle = 10°, repetition time (TR) = 3.85 msec, and echo time (TE) = 2.46 msec. Following the µ-map acquisition, the 18F-fluorocholine PET tracer (BV Cyclotron VU, Amsterdam, the Netherlands) was intravenously injected with a dose of 4 MBq/kg (up to a maximum dose of 360 MBq). Five minutes after the PET tracer injection, a static list-mode PET acquisition was started while the respiratory signal was recorded using the respiratory belt. Previous research in the carotid artery showed stable FCH uptake from 10 min until 1 h after injection in both symptomatic and asymptomatic carotid arteries, as well as in vascular background [15].

Simultaneously, during PET acquisition, coronary MR angiography (CMRA) was performed 2 min after an intravenous contrast agent injection of 0.2 mmol/kg gadobutrol (Gadovist; Bayer Pharmaceuticals, Berlin, Germany), up to a maximum of 20 mmol. A 3D spoiled gradient-echo sequence with a fully sampled golden-step Cartesian trajectory with spiral profile ordering was used. Relevant sequence parameters include the following: FOV = 304 × 304 × 104 mm^3^, acquired resolution = 1.0 × 1.0 × 2.0 mm^3^, flip angle = 15°, TR = variable based on heart rate, TE = 1.7 msec, and acquisition window ranging between 90 and 130 msec. Three-lead ECG registration was performed to allow for cardiac motion gating. Every heartbeat, just before each acquisition window, a 2D image navigator (iNAV) was acquired, which provides a low-resolution image of the heart in coronal view to allow for subsequent motion correction. Details of this sequence have been previously described [19].

### 2.3. OCT Imaging

Within 72 h after the hybrid PET-MRI scan, during the planned second PCI procedure at the VieCuri hospital in Venlo, OCT imaging of the secondary pathological vessel was performed before potential stent placement. Before entry into the coronary artery, an intracoronary injection of 100 to 200 µg nitroglycerine was provided. The tip of the OCT catheter (Dragonfly intravascular imaging catheter, St. Jude Medical, St. Paul, MN, USA) was then placed at least 5 mm distal to the distal edge of the lesion. While obtaining optimal blood clearance by flushing the coronary artery with contrast agent using an automated pump, an automatic pullback through the lesion was initiated, covering at least 5 mm of the proximal and distal parts of the vessel.

### 2.4. Image Reconstruction

PET image reconstruction was performed with e7 Tools (Siemens Healthineers, Erlangen, Germany) using the ordinary Poisson-ordered subset expectation maximization (OP-OSEM) algorithm with 3 iterations and 21 subsets. Images were reconstructed with a voxel size of 2.08 × 2.08 × 2.03 mm^3^ and a matrix size of 344 × 344 × 127. For PET attenuation correction, MR-based Dixon µ-maps were used that provided separation between air, lung, fat, and soft tissue. To make up for the smaller FOV of MRI with respect to PET, the maximum likelihood reconstruction of attenuation and activity (MLAA) approach was utilized.

For CMRA motion correction, motion in the left-right (LR) and feet-head (FH) direction is estimated for each heartbeat using the apex of the heart in the iNAV images. Based on the amplitude in the FH direction, CMRA data is allocated to four respiratory phases or bins. The k-space data inside each bin is corrected to the center of the bin using the FH and LR position estimates derived from the iNav. Next, each bin is reconstructed. Using the end-expiration bin as a reference, respiratory non-rigid deformation fields are generated, which are subsequently applied to transform each bin to the end-expiration position, generating the motion-compensated CMRA image [19].

Three different motion-correction strategies were available to correct the PET datasets for respiratory motion: (1) Multiphase respiratory gating motion correction (MRG-MOCO), where motion correction was based solely on the respiratory belt signal as acquired during the entire PET acquisition. Only the PET data acquired during the end-expiration phase were used for image reconstruction. (2) Extended MR-based MOCO (eMR-MOCO), where both the iNav respiratory signal (as described earlier, but only available during ~9 min of CMRA imaging) and the respiratory belt signal (available entire PET acquisition) are used [20]. The time window for which both the iNav respiratory signal and the respiratory belt signal were collected was used to ensure that the binning of PET data on the iNav signal closely matches the binning on the respiratory belt signal by adjusting binning thresholds. These thresholds are then extended for respiratory motion correction of the complete duration of the PET scan. Each bin was reconstructed and combined with other bins using iNav-based motion fields to a reference position to create a respiratory motion-corrected dataset. (3) Finally, eMR-MOCO-ECG applied the combination of eMR-MOCO (strategy 2) and ECG-based cardiac gating to mitigate cardiac motion as well. Only the PET data acquired during the end-diastolic phase, which was derived from the CMRA sequence, was used for eMR-MOCO-ECG reconstruction; other data was discarded. An overview of the three motion-correction strategies is provided in Table 1.

### 2.5. Image Analysis

The OCT data was analyzed by an independent core lab (LIMIC Medical, Ridderkerk, The Netherlands), where fibrous cap thickness was determined in all plaques. Plaque vulnerability was defined as a plaque with a thin fibrous cap of ≤70 μm [16]. PET imaging was then co-registered with the CMRA images and analyzed using MIM Vista version 7.3.7 (MIM software, Cleveland, OH, USA). The OCT slice position of the vulnerable plaque was located on the 3D MRI, using vessel side branches as landmarks, by a cardiologist (BR) and nuclear medicine physician (JP), blinded to PET images. A volume of interest (VOI) was defined around the pathological vessel section. In the same vessel, a control lesion with a thick fibrous cap was selected on OCT, pinpointed on the MRI, and analyzed using the same approach as described for the target lesion. After defining the vessel section VOIs, the PET imaging was revealed in the PET and fusion viewports. At this point, the VOIs were only altered when there was an overspill of activity from neighboring structures. The maximum standardized uptake value (SUVmax) was measured in both the target and control lesions. Target-to-background ratios (TBRs) were calculated by dividing SUVmax values of the target and control lesions by the mean standardized uptake value (SUVmean) of the blood pool in the left atrium.

### 2.6. Statistics

Differences in TBRs between vulnerable and stable plaques were tested by paired Student’s *t*-test (normally distributed data) or Wilcoxon signed-rank test (non-normally distributed data). Normality of data was tested using the Shapiro–Wilk test. The correlation between TBRmax and minimal fibrous cap thickness for the combined target and reference lesions was assessed using Spearman’s rho correlation coefficient for each PET reconstruction. All statistical analyses and plot generations were performed using RStudio (Integrated Development Environment for R, version 1.4.1103, Boston, MA, USA). Two-tailed values of *p* < 0.05 were considered significant.

## 3. Results

**Figure 1:**
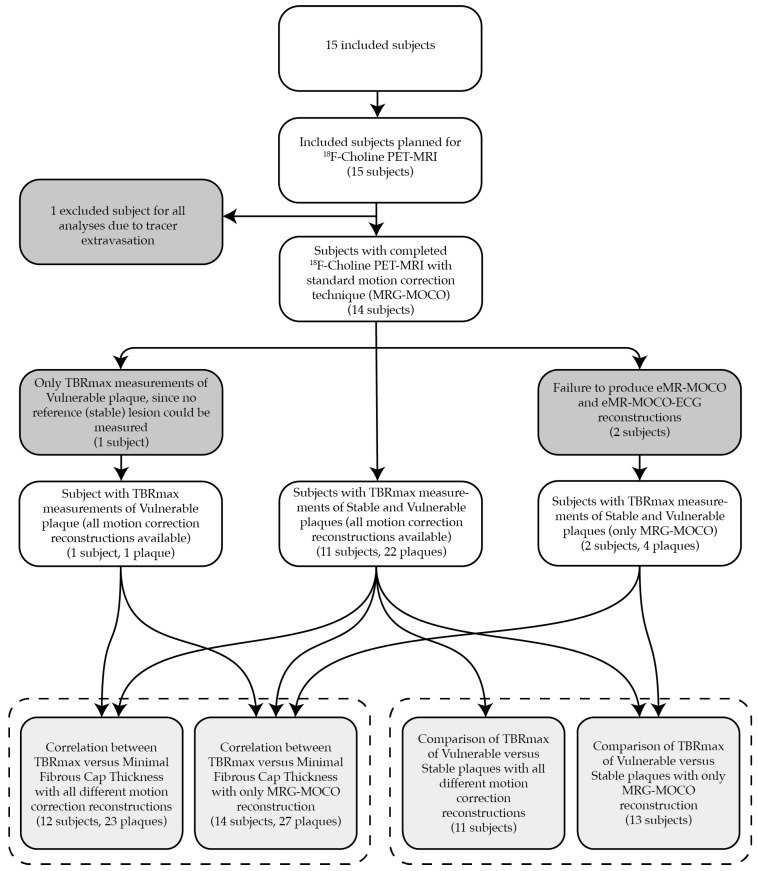
This flowchart provides an overview of the reasons for partial or total exclusion of subjects in the different analyses. In one subject, the PET-MRI data could not be used because of tracer extravasation. In three other subjects, data was partially used, as depicted in the flowchart. The bottom row of boxes resembles the actual analyzed data, grouped per analysis by the dashed box, specifying the number of subjects for each subanalysis, as well as the number of plaques for the TBRmax versus minimal fibrous cap thickness correlation analysis.

A total of 15 patients were included in this study. One subject was excluded from the final analysis due to PET tracer extravasation. The baseline characteristics of the remaining 14 patients are described in Table 2. Three subjects could only be partially analyzed for the following reasons: (1) CMRA with nondiagnostic image quality due to artifacts (excluded for TBRmax eMR-MOCO and eMR-MOCO-ECG analyses), (2) no coronary reference stable lesion available without a thin fibrous cap in a diffusely diseased vessel (*n* = 1; excluded for target versus reference TBRmax comparison), and (3) failure to produce eMR-MOCO and eMR-MOCO-ECG respiratory gating reconstructions (excluded for eMR-MOCO and eMR-MOCO-ECG analyses). Figure 1 provides a flowchart with an overview of the reasons for partial or total exclusion of subjects in the different analyses.

**Figure 2:**
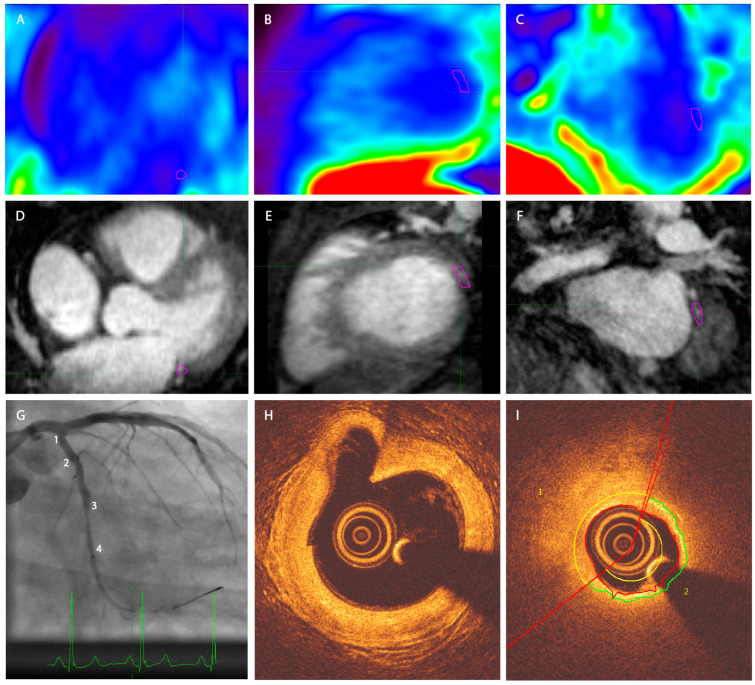
Exemplary PET-MRI and OCT image of one patient. (**A**–**C**) Axial, sagittal, and coronal PET. (**D**–**F**) Axial, sagittal, and coronal MRI, (**G**) angiography. Numbers 1–4 represent four different angiographical landmarks to correlate with OCT. A significant stenosis is present between landmarks 3 and 4. (**H**) OCT image corresponding to landmark 1 in panel (**G**), a large bifurcating vessel can be distinguished in the left upper quadrant of the image; (**I**) OCT image in the region of the stenosis as displayed in panel (**G**). The vessel segment between 2 and 3 contains a plaque with a thin cap on the OCT analysis. The purple VOI in panels (**A**–**F**) delineates the target plaque in the left circumflex artery of this patient. No increased tracer uptake was visually detectable in the coronary arteries.

**Figure 3:**
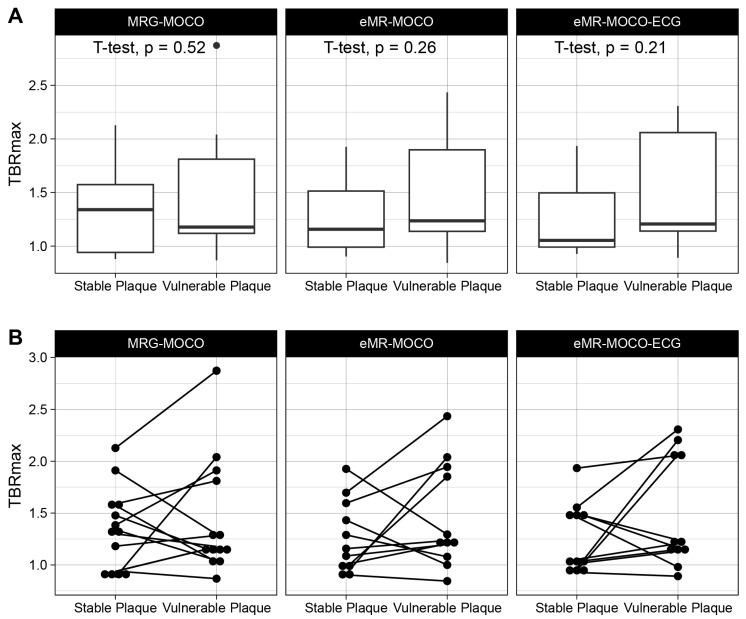
Boxplots (**A**) and dot plots (**B**) of TBRmax values of target and reference lesions. No statistically significant differences were found. The lower and upper hinges of the boxplots represent 25th and 75th percentiles, respectively, and whisker endpoints represent minimum and maximum non-outlying values. The dot in panel (**A**) represents an outlier.

All analyzed patients had a vessel section with a fibrous cap thinner than 70 µm. Figure 2 shows imaging examples of the acquired PET-MRI and OCT imaging. No significant difference between TBRmax values of the vulnerable versus the stable lesions for MRG-MOCO, eMR-MOCO, and eMR-MOCO-ECG was found (MRG-MOCO: mean TBRmax 1.45 vs. 1.35, *p* = 0.52; eMR-MOCO: 1.47 vs. 1.27, *p* = 0.26; eMR-MOCO-ECG: 1.49 vs. 1.26, *p* = 0.21). Boxplots and dot plots of vulnerable versus stable plaque TBRmax values for the different PET reconstructions are shown in Figure 3.

**Figure 4:**
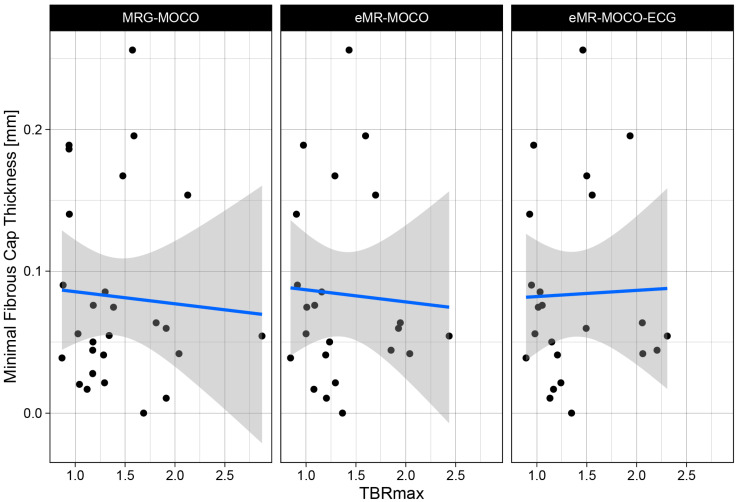
Scatter plot of TBRmax values plotted versus minimal fibrous cap thickness for three different reconstruction methods. No correlation is observed between the TBRmax and the fibrous cap thickness. Regression lines are plotted in blue, with standard error interval displayed as gray area.

No significant correlation between TBRmax and minimal fibrous cap thickness was found for each of the PET reconstructions (MRG-MOCO: −0.034, *p* = 0.87; eMR-MOCO: −0.057, *p* = 0.80; and eMR-MOCO-ECG: −0.036, *p* = 0.87). Scatterplots of the TBRmax and minimal fibrous cap thickness of the combined vulnerable and stable plaques for each PET reconstruction are shown in Figure 4.

**Figure 5:**
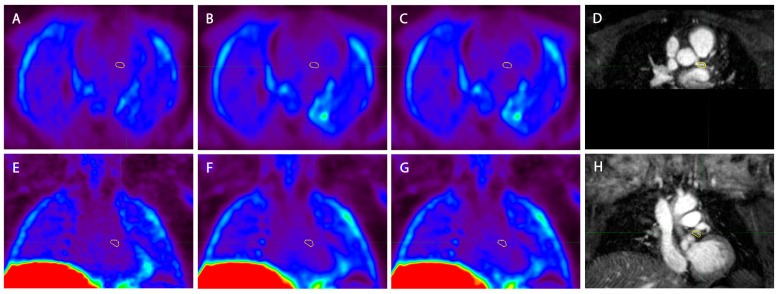
Example of the three different PET reconstructions in one patient (**A**) and (**E**) standard multigate respiratory gating motion correction (MRG-MOCO), (**B**) and (**F**) extended MR-based motion correction (eMR-MOCO), (**C**) and (**G**) extended MR-based motion correction with ECG gating (eMR-MOCO-ECG), accompanied by 2D image-navigator-based motion-corrected 3D whole-heart MRI for anatomical reference (**D**) and (**H**). The top row images show axial reconstructions, while the bottom row shows coronal reconstructions. The yellow volume of interest (VOI) was drawn over the main branch of the left coronary artery (**A**–**H**), which contains a vulnerable plaque. Within the VOI, no visually increased uptake was observed.

A comparison of the three used PET imaging reconstructions is shown in Figure 5. A difference in noise can be visually appreciated between the different reconstructions, where the MRG-MOCO reconstruction yields more noise than both eMR-MOCO and eMR-MOCO-ECG.

## 4. Discussion

To the best of our knowledge, this is the first study that investigates the feasibility of identifying vulnerable coronary plaques with non-invasive 18F-fluorocholine PET. Coronary imaging is challenging due to inherent motion artefacts caused by both respiratory and cardiac motion. To correct for these motion artifacts, advanced hybrid PET-MRI respiratory motion correction, as well as cardiac gating methods, were applied in different combinations. Nevertheless, no significant differences were found in 18F-fluorocholine PET uptake between vulnerable and stable coronary plaques as classified by invasive OCT imaging as a reference standard. The use of hybrid PET-MRI in the present study allows for non-rigid respiratory motion correction in our patient population. This PET-MRI reconstruction method allowed us to estimate translational motion from a low-resolution 2D MR image navigator (iNav) acquired each heartbeat and to subsequently apply non-rigid respiratory motion correction between different respiratory bins from the CMRA data. In contrast, respiratory motion-correction methods in PET-CT for the correction of pulmonary and upper abdominal motion artifacts are only correct in the craniocaudal direction. This important advantage of hybrid PET-MRI potentially provides a non-invasive method for analyzing the coronary arteries. Another advantage is that molecular imaging on PET can be combined with functional ischemia imaging on MRI.

The advanced non-rigid respiratory motion-correction techniques that were used in the present study (eMR-MOCO and eMR-MOCO-ECG) correct for respiratory motion in the feet-head and left-right directions, without discarding data. Although we regard this as an important technological step forward, a small amount of residual motion in the anterior–posterior direction can still be present. We regard this as a limitation of the current study, since techniques for motion correction in three dimensions were not available. The MRG-MOCO and eMR-MOCO reconstructions were not corrected for cardiac motion, which leads to motion-induced image blurring. Therefore, we applied ECG-gating with the eMR-MOCO-ECG reconstruction. Also, for this fully motion-corrected reconstruction, no difference in 18F-fluorocholine PET uptake between vulnerable and stable coronary plaques was found.

In contrast to 18F-FDG PET imaging, which has significant drawbacks for coronary imaging, radiolabeled choline is highly taken up in activated macrophages and was hypothesized to be an alternative, more specific tracer for imaging plaque inflammation [15]. Initial murine and rabbit models of atherosclerosis revealed a significantly higher uptake of radiolabeled choline in inflamed atherosclerotic plaques in comparison to healthy vessel wall [21,22,23]. This rapid uptake of radiolabeled choline in plaque macrophages seems to be linked to the upregulation of choline transporters on the cell surface, similar to that observed in macrophages from other inflammatory conditions [14,22]. There are three published reports that retrospectively analyzed choline PET in the diagnosis of prostate cancer, and described a higher presence of the tracer within the atherosclerotic vessel walls [24,25,26]. For obvious reasons, no comparison with the gold standard, histology, has been made in these studies. Our own group earlier investigated vascular wall inflammation in a prospective study that compared 18F-fluorocholine uptake to macrophage content on histology, represented by CD68+ plaque content, in a patient population of symptomatic carotid artery stenosis [15]. That study showed a positive correlation between 18F-fluorocholine uptake and macrophage content, as well as higher uptake in the ipsilateral versus contralateral carotid vessel wall.

An explanation for the lack of 18F-fluorocholine uptake in vulnerable coronary plaques in the current study could be related to the small size of the coronary plaques, compared to larger carotid artery lesions studied earlier. A certain minimum amount of tracer needs to accumulate in a plaque in order to be detected by PET. Sub-voxel size of plaques does not rule out detection, but the partial volume effect can negate detectability. Studies with other tracers, notably 18F-sodiumfluoride (18F-NaF), revealed increased tracer uptake in culprit versus non-culprit coronary plaques of patients with myocardial infarction [27]. Interestingly, this study also showed that patients with 18F-NaF-positive plaques in a stable angina cohort had higher 18F-NaF activity when compared to those with myocardial infarction. The patients with stable angina were older and, therefore, may have had a more extensive plaque burden. Thus, not only the vulnerability but also the size of the plaque determines the TBRmax value. Another difference between coronary and carotid PET imaging is that the carotid arteries are not affected by respiratory motion and cardiac motion, which makes the carotid PET image substantially less challenging.

When investigating coronary artery plaques, no coronary artery specimens can be obtained to correlate PET outcomes to histological findings. In our study, invasive OCT imaging was used as a reference standard to provide detailed images of coronary plaques with a resolution ranging from 10 to 20 µm. This is approximately 50–100 times higher than what can be achieved using MRI or intravascular ultrasound (IVUS), and OCT has been well validated in numerous studies [16,17,28,29,30]. The OCT parameter that we used to define a vulnerable plaque was the presence of a thin fibrous cap, since rupture of such a cap is the main underlying cause of myocardial infarction. We used a threshold of a cap thickness of less than 70 µm for a vulnerable plaque in line with previous studies [31]. While pathologists define a thin fibrous cap using a cut-off value of <65 µm [32], for OCT, one should choose a slightly higher threshold to take into account tissue shrinkage during histological processing [33].

A limitation of this study is that we only used a fibrous cap thickness threshold of less than 70 µm as a criterion for plaque vulnerability. While other characteristics, which are associated with plaque vulnerability, such as lipid burden and macrophage features, can be derived from OCT, we preferred to use a single parameter in order to have a clear parameter that unequivocally identifies vulnerable plaques versus stable plaques in this study with a small sample size.

The anatomical correlation of the plaques on OCT and PET relied on visually comparing vascular anatomy as displayed on angiographical imaging to the coronary MR angiography images. By carefully selecting a segment with stable plaque, sufficiently distant from vulnerable plaques, we ensured that the stable plaque VOI did not include a section of vulnerable plaque. We also made sure that the entire plaque was included in the VOI. The advantage of TBRmax is that this parameter is not influenced by the VOI size, as long as no other 18F-fluorocholine avid structures are included. Therefore, the current negative results cannot be explained by high uptake in stable plaques. Theoretically, a cone-beam CT acquired using a state-of-the-art angiographic gantry could be better suited to co-register the OCT pullback trajectory with the PET images. On the other hand, such a technique would lead to extra radiation exposure.

In the present study, we included NSTEMI patients, where myocardial infarction typically induces a systemic inflammatory response that could influence plaque inflammation and tracer uptake. However, since we performed a paired comparison of tracer uptake between stable and vulnerable plaques within the same patient, the potential effect of the time between NSTEMI and the PET-MRI scan is likely to be minimized. Both stable and vulnerable plaques in the same subject would be similarly affected by the systemic inflammatory response, making the timing less critical for assessing relative differences in tracer uptake between these plaques. Furthermore, there was a considerable amount of time of an average of 30 days, between NSTEMI and PET-MRI, with limited variation in this interval between patients.

A practical limitation concerning the analysis of 18F-fluorocholine uptake in coronary vessels is intense liver uptake, obscuring any vascular uptake located close to the liver due to spill-over of liver activity. This mostly affects the distal right coronary artery, where the lumen and plaques tend to be smaller and are already inherently more challenging for PET imaging in general. In our patient population, however, all vulnerable and stable plaques that were analyzed were in regions not susceptible to activity overspill from the liver. Another limitation of this study is the small sample size since the study was conceived to establish proof of principle. Therefore, this study is not fully able to test diagnostic performance. Although a study with a larger sample size might have been able to detect a statistically significant difference between the TBRmax in vulnerable versus stable lesions, we found that there was a high overlap between the tracer uptake in vulnerable and stable plaques. In approximately one-third of the patients, even a higher TBRmax was observed in the stable than in the vulnerable plaque. Therefore, it is unlikely that a study with a higher sample size will find clinically relevant differences.

In recent years, the perivascular fat attenuation index as measured with CTA has emerged as a marker for coronary inflammation [34]. Although the perivascular fat attenuation index is an indirect marker, while the uptake of a PET tracer in a plaque is a direct marker of plaque inflammation, the perivascular fat attenuation index can be quantified on CTA, which is much more widely available compared to PET and comes with a lower ionizing radiation dose. In the same CTA examination also other CTA imaging risk markers could also be obtained, such as low-attenuation coronary plaque, luminal stenosis severity, coronary calcium score, and total plaque volume [35].

## 5. Conclusions

PET-MRI with 18F-fluorocholine, using advanced respiratory non-rigid motion correction with or without cardiac gating, did not show significantly higher uptake in vulnerable plaques compared to stable plaques using OCT as a reference standard. These findings in the coronary arteries differ from previous results in the carotid arteries, where more uptake in symptomatic lesions was observed. Multiple inherent factors, such as small coronary plaque size, tracer biology, and cardiac movement, may hamper visualization of coronary macrophage plaque content through 18F-fluorocholine binding.

## Figures and Tables

**Table 1:** Overview of the three motion-correction strategies.

	MRG-MOCO	eMR-MOCO	eMR-MOCO-ECG
**Respiratory motion-correction method**	PET reconstruction based on data acquired during end-expiration, as determined using the respiratory belt	Respiratory motion correction based on iNav-based respiratory motion fields	Respiratory motion correction based on iNav-based respiratory motion fields
**ECG gating**	No	No	Yes (end-diastolic phase)

MRG-MOCO: multiphase respiratory gating motion correction; eMR-MOCO: extended MR-based MOCO; eMR-MOCO-ECG: eMR-MOCO and ECG-based cardiac gating, iNav: image based navigator.

**Table 2:** Baseline subject characteristics.

Characteristic	All Subjects (n14)
Age [years]	63.1 (10.3)
Gender (female; *n*,%)	3 (21.4)
Weight [kg]	81.3 (12.0)
Systolic blood pressure [mmHg]	135.2 (22.7)
Diastolic blood pressure [mmHg]	72.4 (11.7)
Hemoglobin [mmol/L]	8.5 (1.1)
LDL-cholesterol [mmol/l]	3.2 (1.6)
HDL-cholesterol [mmol/L]	1.1 (0.27)
Total cholesterol [mmol/L]	4.7 (2.0)
Triglyceride [mmol/L]	1.5 (0.6)
Hypertension (*n*,%)	6 (42.9)
Diabetes mellitus (*n*,%)	2 (14.3)
Hypercholesterolemia (*n*,%)	6 (42.9)
Obesity (*n*,%)	4 (28.6)
Smoking (*n*,%)	
Never smoked	3 (21.4)
Former smoker	4 (28.6)
Quit smoking during current hospitalization	5 (35.7)
Still smoking	2 (14.3)
History of ischemic heart disease (*n*,%)	1 (7.1)
Antihypertensive agent use at intake (*n*, %)	2 (14.3)
Antihypertensive agent use at discharge (*n*, %)	13 (92.9)
Antiplatelet agent use at intake (*n*,%)	0 (0)
Antiplatelet agent use at discharge (*n*,%)	14 (100)
Oral anticoagulant use at intake (*n*,%)	0 (0)
Oral anticoagulant use at discharge (*n*,%)	2 (14.3)
Cholesterol synthesis inhibitor use at intake (*n*,%)	2 (14.3)
Cholesterol synthesis inhibitor use at discharge (*n*,%)	14 (100)
Oral antidiabetic use at intake (*n*,%)	1 (7.1)
Oral antidiabetic use at discharge (*n*,%)	2 (14.3)
Parenteral antidiabetic use at intake (*n*,%)	0 (0)
Parenteral antidiabetic use at intake (*n*,%)	0 (0)
Time interval between NSTEMI and PET-MRI [days] (mean (SD))	30.1 (6.9)

All data are indicated as mean (standard deviation), unless indicated otherwise. Medication use was recorded at intake as well as discharge, during first hospitalization for the NSTEMI event.

## Data Availability

The data presented in this study are available on request from the corresponding author on reasonable request.

## Abbreviations

The following abbreviations are used in this manuscript:

BMI	Body Mass Index
CD68	Cluster of Differentiation 68
CMR	Cardiac Magnetic Resonance
CMRA	Coronary Magnetic Resonance Angiography
CTA	Computed Tomography Angiography
18F-FDG	18-Fluor-fluorodeoxyglucose
eMR-MOCO	Extended MR-Based Motion Correction
eMR-MOCO-ECG	Extended MR-Based Motion Correction with ECG gating
FDG	18F-fluorodeoxyglucose
FOV	Field Of View
GRACE-score	Global Registry of Acute Coronary Events score
ICA	Invasive Coronary Angiography
iNAV-CASPR-MOCO	2D Image Navigator-Based 3D Whole-Heart Sequence Cartesian Trajectory with Spiral Profile Motion Corrected
MI	Myocardial Infarction
MRI	Magnetic Resonance Imaging
MRG-MOCO	Multigate Respiratory Gating Motion Correction
NSTEMI	Non-ST-Segment Elevation Myocardial Infarction
OCT	Optical Coherence Tomography
PCI	Percutaneous Coronary Intervention
PET	Positron Emission Tomography
STEMI	ST-Segment Elevation Myocardial Infarction
SUVmax	Maximal Standardized Uptake Value
SUVmean	Mean Standardized Uptake Value
TBRmax	Maximal Target-to-Background Ratio
VOI	Volume of Interest
